# Harnessing online digital data in biodiversity monitoring

**DOI:** 10.1371/journal.pbio.3002497

**Published:** 2024-02-15

**Authors:** Andrea Soriano-Redondo, Ricardo A. Correia, Vijay Barve, Thomas M. Brooks, Stuart H. M. Butchart, Ivan Jarić, Ritwik Kulkarni, Richard J. Ladle, Ana Sofia Vaz, Enrico Di Minin

**Affiliations:** 1 Helsinki Lab of Interdisciplinary Conservation Science (HELICS), Department of Geosciences and Geography, University of Helsinki, Helsinki, Finland; 2 Helsinki Institute of Sustainability Science (HELSUS), University of Helsinki, Helsinki, Finland; 3 Biodiversity Unit, University of Turku, Turku, Finland; 4 Marine Biodiversity Center, Natural History Museum of Los Angeles County, Los Angeles, California, United States of America; 5 International Union for Conservation of Nature (IUCN), Gland, Switzerland; 6 World Agroforestry Center (ICRAF), University of the Philippines Los Baños, Laguna, Philippines; 7 Institute for Marine and Antarctic Studies, University of Tasmania, Hobart, Tasmania, Australia; 8 BirdLife International, Cambridge, United Kingdom; 9 Department of Zoology, University of Cambridge, Cambridge, United Kingdom; 10 Université Paris-Saclay, CNRS, AgroParisTech, Ecologie Systématique Evolution–IDEEV, Gif-sur-Yvette, France; 11 Biology Centre of the Czech Academy of Sciences, Institute of Hydrobiology, České Budějovice, Czech Republic; 12 Instituto de Ciências Biológicas e da Saúde, Universidade Federal de Alagoas, Maceió, Brazil; 13 CIBIO, Centro de Investigação em Biodiversidade e Recursos Genéticos, InBIO Laboratório Associado, Campus de Vairão, Universidade do Porto, Vairão, Portugal; 14 Departamento de Biologia, Faculdade de Ciências, Universidade do Porto, Porto, Portugal; 15 BIOPOLIS Program in Genomics, Biodiversity and Land Planning, CIBIO, Campus de Vairão, Vairão, Portugal; 16 School of Life Sciences, University of KwaZulu-Natal, Durban, South Africa

## Abstract

Online digital data from traditional and social media platforms has the potential to complement biodiversity monitoring efforts. In this Perspective, the authors propose a strategy for integrating these data into current biodiversity datasets.

## Teaser

Online digital data from media platforms have the potential to complement biodiversity monitoring efforts. We propose a strategy for integrating these data into current biodiversity datasets in light of the Kunming-Montreal Global Biodiversity Framework.

Biodiversity is declining at unprecedented rates, which is limiting our capacity to respond to other sustainability challenges, including climate change. Addressing the biodiversity crisis requires collecting accurate, large-scale data for monitoring the status of species, habitats, and ecosystems over time and space. Without large-scale biodiversity monitoring, our capacity to investigate the human activities that drive biodiversity loss or that support nature conservation and restoration would be severely limited, with catastrophic consequences for nature conservation and people [1]. These requirements are reflected in the Kunming-Montreal Global Biodiversity Framework, an international agreement that sets out a pathway to achieve harmonious coexistence with nature by 2050. However, biodiversity monitoring suffers from significant taxonomic and geographic gaps owing to inequalities in resources and capacity [2]. To address this shortcoming, we propose the strategic use of the enormous volume of available online biodiversity data (originally generated for purposes other than biodiversity conservation) [3], as a cost-efficient method for monitoring biodiversity and human activities.

Such online digital data can be used to strengthen existing assessments of the status and trends of biodiversity, the pressures upon it, and the conservation solutions being implemented, as well as to generate novel insights about these aspects, nature’s contributions to people, and human–nature interactions [3,4]. The most common sources of online biodiversity data include web pages, news media, social media, image- and video-sharing platforms, and digital books and encyclopedias [5]. These data can be filtered and processed by researchers to target specific research questions and are increasingly being used to explore ecological processes and to investigate the distribution, spatiotemporal trends, phenology, ecological interactions, or behavior of species or assemblages and their drivers of change [3].

Digital data are also being used to explore human–nature interactions from multiple perspectives. For example, photographs from Flickr have been used to explore cultural ecosystem services, Twitter/X or YouTube have been used to identify instances of illegal wildlife trade, and Wikipedia page-views have been used to assess observable patterns in seasonal phenomena of plants and animals [5]. Such diversity of uses showcases the great potential of these data to provide novel insights into nature and human–nature interactions, and to track global biodiversity trends through continued monitoring. However, it also shows the unstructured nature of this area of research, as researchers mostly select digital media platforms ad hoc. Moreover, digital data are not currently integrated into standardized biodiversity monitoring programs, thereby preventing a streamlined utilization of these digital media resources for biodiversity conservation.

**Fig 1:**
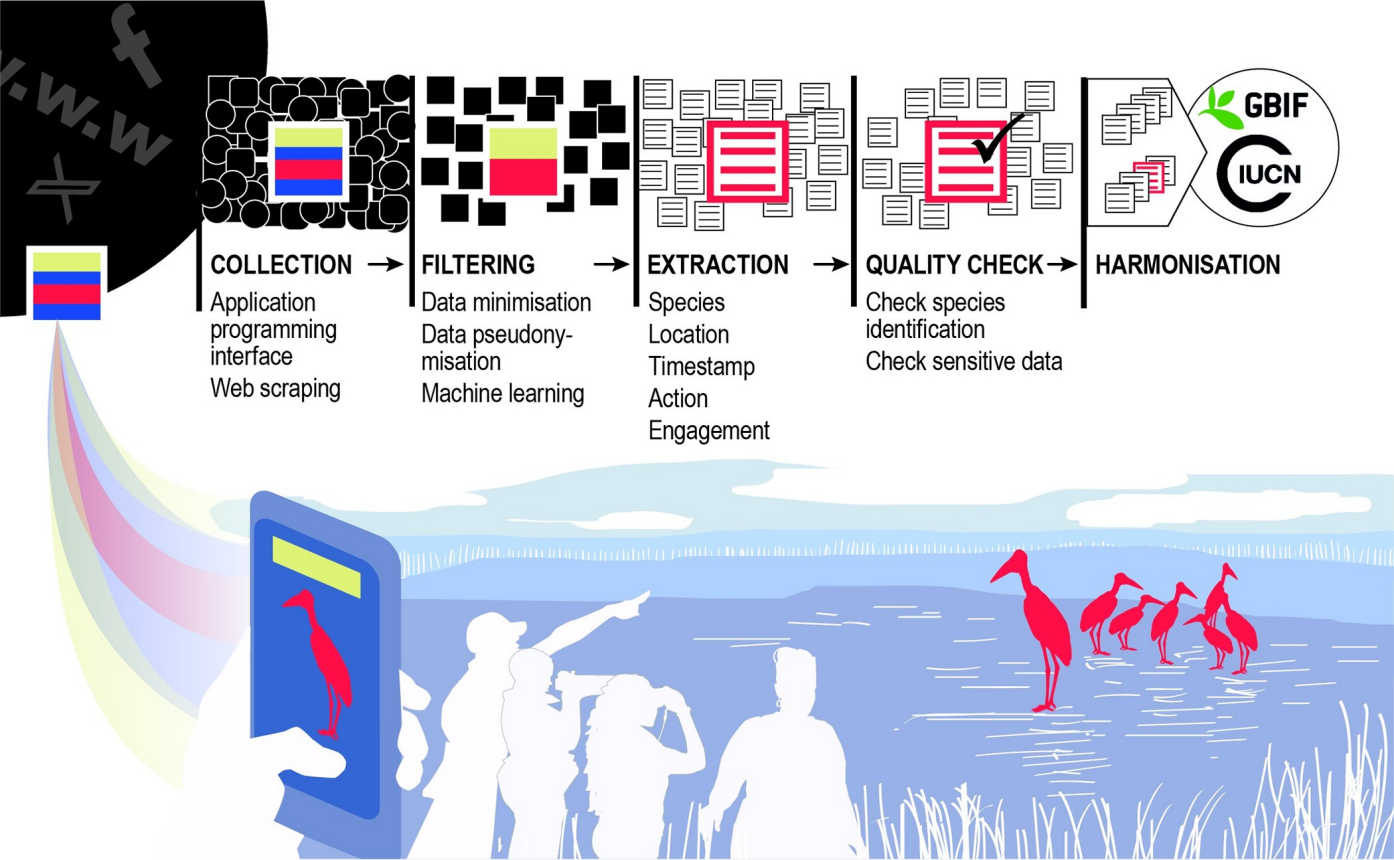
Proposed framework for integrating online digital data into biodiversity monitoring. Workflow of the proposed framework, from data collection to data harmonization.

In support of the Kunming-Montreal Global Biodiversity Framework targets and the UN Sustainable Development Goals, we are calling for urgent action to identify, collect, filter, extract, store, integrate, share, and disseminate digital online data to better capture biodiversity status and trends in near real-time (Fig 1). Such data include complementary text, image, video, and/or sound content pertaining to the natural world that is uploaded into the digital realm, and that can be integrated into current datasets and monitoring schemes on biodiversity, from individual organisms to whole ecosystems, and the way these dimensions intersect with human society [5].

To collect meaningful digital data for biodiversity monitoring, we propose an approach that follows standard assessments of species (e.g., the International Union for Conservation of Nature (IUCN) Red List of Threatened Species), ecosystems (e.g., the IUCN Red List of Ecosystems [6]), and nature’s contributions to people [7], as well as classification schemes for threats and conservation actions (e.g., Unified Classifications of Threats and Actions [8]). The approach would consist of continually searching and retrieving data for a selected list of keywords or topics that are particularly relevant to complement current data streams, through direct scraping or via dedicated open Application Programming Interfaces (APIs) of social media platforms or search engines. For example, a set of keywords could include the list of names of all described species in multiple languages.

Moving forward, the collection of online digital data will require filtering and the extraction of relevant information for biodiversity from large corpora, and hence the construction of automated pipelines that can leverage developments in machine learning methods (Fig 1). To filter data from digital text, these pipelines would need to deduplicate information and remove irrelevant entries using, for example, text vectorization algorithms and artificial neural networks [9]. Named entity recognition could then be used to extract specific data such as the name of a species, timestamp, geographic coordinates, and/or other quantifiable information (e.g., quantities and prices) to be included in structured biodiversity datasets. Classifying information from digital images will follow a similar process, as it requires the implementation of machine vision models to tease apart relevant and irrelevant images [10].

One of the major limitations of these methods is that they require the creation of high-quality labeled training datasets covering all the necessary content. An additional challenge in the case of text is the global diversity of languages, which would necessitate multilingual models. Moreover, after extraction, data would require automated validation and flagging of uncertain records to minimize erroneous information entering the data stream. Machine learning techniques could be used to partially overcome some of the limitations, such as the uneven coverage of digital data across regions, time, taxa, and ecosystems (for example, areas with low human population densities are likely to be underrepresented in the datasets and charismatic species to be overrepresented) by extrapolating some of the missing data.

Following the proposed framework, only the specific data required to monitor biodiversity would be collected and stored, the rest would be discarded, and any information that could be used to identify individuals would be pseudonymized [11]. Data minimization and pseudonymization principles would be included and enforced in the pipelines to minimize the risk of leaking sensitive information. Publication of data derived from online sources would also need to take account of recognized best practices in minimizing risk of harm to biodiversity posed by revealing the precise locations of highly threatened or valuable species [12].

Data generated through the framework in near real-time could be continuously integrated with other independently collected datasets and used for real-time applications; for example, as part of integrated spatial conservation planning assessments and adaptive management (Fig 1). Species occurrence data could be collected following Biodiversity Information Standards, such as the Darwin Core Standard, and uploaded into the Global Biodiversity Information Facility (GBIF). Data relevant to assessment of species extinction or ecosystem collapse risk, perhaps most usefully relating to threats, could be mobilized into the workflows for generating the IUCN Red List of Threatened Species and Red List of Ecosystems, respectively, while data on sites of global significance for the persistence of biodiversity (and the occurrence of species within them) could be served to the appropriate national coordination groups to strengthen their efforts in identifying Key Biodiversity Areas. Moreover, data on the illegal wildlife trade could be integrated with the Convention on International Trade in Endangered Species of Wild Fauna and Flora (CITES) Trade Database or the Trade Records Analysis of Flora and Fauna in Commerce (TRAFFIC) open-source wildlife seizure and incident data.

The necessary technology to implement this framework is within reach; it only requires willingness and resources to put it into action. Reaching the full potential of the framework will require harnessing expertise from multiple sectors and academic disciplines, as well as the collaboration of digital media companies. The proprietary companies that own social media platforms, search engines, and other digital platforms and their respective APIs, often limit access to their content, either by capping it to a certain volume and/or by requiring paid subscription. Data availability varies greatly depending on the platform, and platform policies can change at any given time, further restricting (or granting) access. Therefore, the full potential of a digital observatory of biodiversity will only be achieved if digital media companies provide researchers with unfiltered access to all relevant content. Furthermore, the long-term sustainability of such a system will require the commitment of an established international and intergovernmental organization to maintain and host the system and update its pipelines, as well as addressing data quality concerns through engagement of expert communities and response to feedback.

The current lack of integration of online digital data into biodiversity monitoring undermines efforts to collect all critical information relevant to identify conservation priorities and implement actions. While such a system should not divert resources from other monitoring efforts, leveraging the ongoing digital data revolution would generate complementary and timely evidence to inform decision-making on the conservation, restoration, and sustainable use of nature and its contributions to people.
